# Mouse Chd4-NURD is required for neonatal spermatogonia survival and normal gonad development

**DOI:** 10.1186/s13072-022-00448-5

**Published:** 2022-05-14

**Authors:** Rodrigo O. de Castro, Agustin Carbajal, Luciana Previato de Almeida, Victor Goitea, Courtney T. Griffin, Roberto J. Pezza

**Affiliations:** 1 Cell Cycle and Cancer Biology Research Program, Oklahoma Medical Research Foundation, Suite B305. 825 NE 13th street, Oklahoma City, OK 73104 USA; 2 Cardiovascular Biology Research Program, Oklahoma Medical Research Foundation, Oklahoma City, OK USA; 3 Department of Cell Biology, University of Oklahoma Health Science Center, Oklahoma City, OK USA

**Keywords:** Mouse gametogenesis, Spermatogonia, Chromatin remodeling, NURD

## Abstract

Testis development and sustained germ cell production in adults rely on the establishment and maintenance of spermatogonia stem cells and their proper differentiation into spermatocytes. Chromatin remodeling complexes regulate critical processes during gamete development by restricting or promoting accessibility of DNA repair and gene expression machineries to the chromatin. Here, we investigated the role of Chd4 and Chd3 catalytic subunits of the NURD complex during spermatogenesis. Germ cell-specific deletion of c*hd4* early in gametogenesis, but not *chd3*, resulted in arrested early gamete development due to failed cell survival of neonate undifferentiated spermatogonia stem cell population. Candidate assessment revealed that Chd4 controls expression of *dmrt1* and its downstream target *plzf*, both described as prominent regulators of spermatogonia stem cell maintenance. Our results show the requirement of Chd4 in mammalian gametogenesis pointing to functions in gene expression early in the process.

**Supplementary Information:**

The online version contains supplementary material available at 10.1186/s13072-022-00448-5.

## Introduction

Defects in gametogenesis are a leading cause of infertility and an important cause of birth defects associated with aneuploidy. Insights into the mechanisms underlying testis formation, including spermatogonia stem cell, are necessary to improve the outcomes of common gonad developmental diseases.

In mice, spermatogenesis begins from isolated germ cells called spermatogonia A-singles (As) that undergo to a series of mitotic divisions to produce spermatogonia known as paired (Apr) and aligned (Aal) that contains chains of 4 to 16 cells anchored by intercellular bridges as a result of incomplete cytokinesis [1, 2]. At this point, spermatogonia cells start a process of differentiation [A1, A2, A3, A4 or intermediate (In)], formation of type B spermatogonia, and then transition to pre-leptotene cells that initiate the series of meiotic divisions that ultimately originate spermatozoa.

Chromatin undergoes extensive remodeling during gametogenesis, leading to altered gene expression and chromosome organization, and ultimately controlling obligatory developmental transitions, such as the conversion from undifferentiated to differentiated spermatogonia, spermatogonia commitment to meiosis, and meiotic progression [3, 4]. These transitions are accompanied by changes in the structural properties of meiotic chromosomes (monitored by Hi-C), ultimately revealing how the chromosome structure influences fundamental meiotic processes, such as recombination and transcription [5]. The NURD (NUcleosome Remodeling and Deacetylase) is a prominent chromatin modifying complex that functions to control gene expression via chromatin remodeling and histone deacetylation [6, 7]. The NURD complex contains two highly conserved and widely expressed catalytic subunits, Chd3/Mi-2α (chromodomain–helicase–DNA-binding 3) and Chd4/Mi-2β, which are members of the Snf2 superfamily of ATPases [6–10]. NURD plays a central role in various developmental and cellular events, such as controlling the differentiation of stem cells, maintaining cell identity, and responding to DNA damage [10–12]. In testis, Chd5 is required for normal spermiogenesis and proper spermatid chromatin condensation [13], while Chd3/4 has been described to localize at the X–Y pseudoautosomal region, the X centromeric region, and then spreads into the XY body chromatin [14, 15]. Although the role of Chd5 has been well defined, the requirements and mechanisms of Chd4 and Chd3 in mouse gametogenesis and testis development is not completely understood. In a recent work, siRNA knockdown of Chd4 in primary cultures followed by spermatogonia transplantation revealed a loss of the regenerative capability of these cells. Interpretation of RNA-seq data obtained from spermatogonia siRNA treated versus control cell cultures revealed global transcription changes, including genes possibly involved in cell self-renewal [16]. Questions remain unanswered regarding the effect of *chd3* and *chd4* deletion in germ cells and testis development.

In this study, we report that testis-specific specific deletion of *chd4* is essential for testis development and sustained germ cell production, while *chd3* deletion results in no apparent phenotype. This germ cell-specific deletion of *chd4* results in the developmental arrest of undifferentiated spermatogonia in neonatal mice progressing to a Sertoli-only phenotype. Our studies of selected Chd4 target genes and subsequent cytological and expression analysis show that Chd4 control *dmrt1* gene expression and downstream targets, such as *plzf*. We propose these results suggest a possible mechanism by which Chd4 contributes to early germ cell development regulating genes that are required for survival/maintenance of spermatogonia cells.

## Results

### *Chd4* expression during mouse germ cell development

**Fig. 1:**
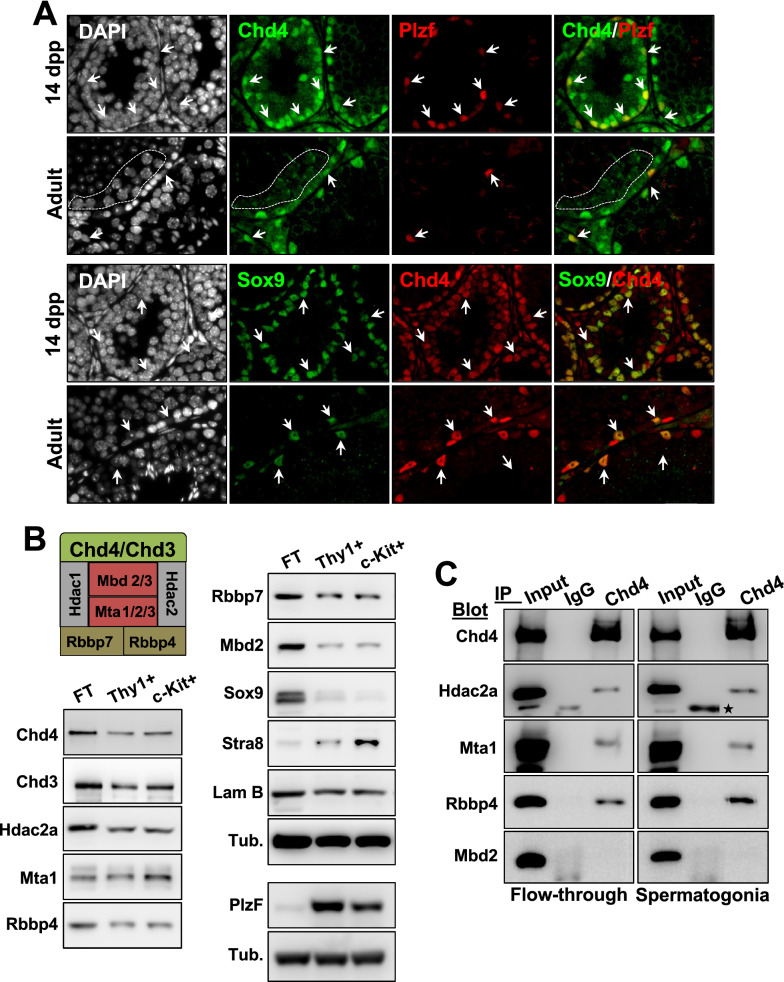
*Chd4* expression and formation of different NURD complexes during gametogenesis. **A** Expression of *chd4* monitored by immunofluorescence in testis sections of 14 dpp and 2 months (adult) old mouse using two distinct antibodies against Chd4, a rabbit polyclonal anti-Chd4 and anti-Plzf (top panel) and mouse monoclonal anti-Chd4 and anti-Sox9 (bottom panel). Arrows indicate examples of Chd4 positive spermatogonia (Plzf) or Sertoli (Sox9) cells. Cells within the punctuated line are pachytene spermatocytes. Similar results were observed independently using three different mice. **B** Western blot analysis of different NURD components in samples of cells enriched in undifferentiated spermatogonia (Thy1.2+) (Plzf positive), differentiating spermatogonia (c-Kit+) (Stra8 positive) and the flow-through (FT), the latter from the spermatogonia enrichment procedure, which contains mostly somatic cells, including a large amount of Sertoli cells (Sox9 positive). Lamin B (Lam B) and α-tubulin (Tub.) were used as loading standards. The scheme represents the composition of a canonical NURD complex. **C** Chd4-participating NURD complexes in enriched fractions of spermatogonia or cells in the flow-through assessed by co-immunoprecipitation with anti-Chd4 as a bait. Non-specific IgG was used as a bait control. Experiments were repeated twice, and the star marks an unspecific reactive band

To investigate a potential role for Chd4 in germ cells, we assessed Chd4 expression in newborn and adult mouse testes by immunofluorescence. Plzf is expressed in undifferentiated spermatogonia [17, 18]. We observed that Chd4 was highly expressed in spermatogonia cells (marked by Plzf, aka Zbtb16) and in Sertoli cells (marked by Sox9) (Fig. 1A and Additional file 1: Fig. S1). In agreement with a previous report [14], immunosignal of Chd4 was detected in late-pachytene stages (Fig. 1A, selected area on adult mouse top panel). In sum, Chd4 is detected in spermatogonia, Sertoli cells, and primary spermatocytes.

We confirmed that *Chd4* is expressed at pre-meiotic stages of male gamete development by analyzing Chd4 protein levels by western blot in enriched fractions (see “Materials and methods” for details) of undifferentiated and differentiating spermatogonia (obtained from wild-type 7 dpp mice and using Thy1.2+ and c-Kit+ affinity columns, respectively) (Fig. 1B). Chd4 was also detected in the affinity column flow-through (FT, which was enriched mostly in Sertoli cells, here demonstrated by immunoblotting of Sox9). The level of cell population enrichment was assessed by western blot and markers specific for undifferentiated spermatogonia (Plzf) and differentiating spermatogonia (Stra8) (Fig. 1B). In addition, we analyzed cell fractions enrichment by immunofluorescence (Additional file 2: Fig. S2).

### Composition of Chd4-NURD complexes during spermatogenesis

NURD function is influenced by its subunit composition [19, 20]. To determine whether the expression of NURD composition might change during spermatogenesis, we analyzed the levels of representative NURD subunits in enriched fractions of undifferentiated and differentiating spermatogonia, as well as in the flow-through (enriched in Sertoli cells) after spermatogonia enrichment. NURD subunits Hdac2a, Mta1, Rbbp4, Rbbp7 and Mbd2 were present in enriched fractions of undifferentiated (Thy1.2+) and differentiating (c-KIT+) spermatogonia (Fig. 1B).

To determine the composition of Chd4-NURD complexes, we used co-immunoprecipitation analysis to uncover NURD subunits that interact with Chd4 in enriched fractions of Thy1.2 and c-Kit spermatogonia cells together and the flow-through. The NURD subunits Hdac2a, Mta1, and Rbbp4/Rbbp7, but not Mbd2, coimmunoprecipitated with Chd4 from both spermatogonia and flow-through fractions (Fig. 1C). Although Mbd2 was enriched in FT (Sertoli) and reduced in spermatogonia fractions (Fig. 1B), we could not observe Mbd2 interaction with Chd4 by immunoprecipitation in any of these two fractions (Fig. 1C). This could be explained because of a transient interaction between Mbd2 and the Chd4-containing NURD complex. Hdac2a coimmunoprecipitated with Chd4 from wild type and c*hd3*^*−/−*^ spermatogonia cells (Additional file 3: Fig. S3B). These data suggest that: (i) Chd4 forms a NURD complex independently of Chd3, (ii) that loss of Chd3 does not perturb Chd4-NURD complex formation in spermatogonia cells.

### Deletion of *chd4* but not *chd3* results in testis developmental defects

**Fig. 2:**
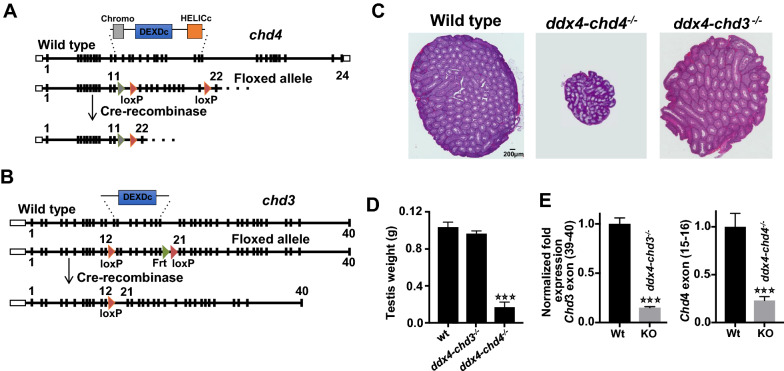
*Chd4* and *chd3* gene targeting design and testis developmental defects in c*hd4* mutant mice. **A**, **B** Testis specific Cre knockout strategy for deletion of *chd4* and *chd3*. See description in “Materials and methods”. **C** H&E-stained paraffin testis sections of wild type, *ddx4-chd4*^*−/−*^*,* and *ddx4-chd3*^*−/−*^ mice. **D** Quantification of testis weight for wild type and homozygous knockout mice is shown. Images and testis weigh measurements were obtained independently from three mice. **E** *Chd3* transcription levels were measured in *ddx4-chd3*^*−/−*^ total testis and compared to wild-type littermates (2 months old mice, *n* = 3). *Chd4* transcription level in enriched fractions of spermatogonia obtained from 7 dpp *ddx4-chd4*^*−/−*^ and control wild-type litter mate mice (mice, biological replicate *n* = 3). *** represents *P* < 0.0001 (two-tailed Student *t* test)

To examine the potential functions of Chd4 and Chd3 during spermatogenesis, we generated *chd4* and *chd3* germline conditional knockout mice (Fig. 2A–E). To delete the floxed allele in gonocytes (embryonic day 15.5) [21], male *ddx4-cre; chd4*^*WT/Δ*^ were crossed with *chd4*^*fl/fl*^ females to generate *ddx4-cre; chd4*^*fl/Δ*^ conditional knockout mice (here called *ddx4-chd4*^−/−^) (Fig. 2A). A similar strategy was used to generate *ddx4-Chd3*^−/−^ mice (Fig. 2B).

We observed that *ddx4-chd4*^*−/−*^ adult mice (2 months old) appeared normal in all aspects except in the reproductive tissues. Testes were significantly smaller in *ddx4-*chd4^−/−^ males (mean: 0.017 g ± SD: 0.005, number of quantified mice *n* = 4 (8 testes), *P* ≤ 0.0001, *t* test) compared to wild type [0.104 g ± 0.015, *n* = 6 mice (12 testes)] littermates (Fig. 2C, D), indicating severe developmental defects in the testis. We confirmed deletion of *chd4* (or *chd3*, later discussed) by RT-qPCR (Fig. 2E). Immunofluorescence analysis of neonatal *ddx4-chd4*^*−/−*^ testis show that Chd4 was absent in Plzf positive spermatogonia at 1 dpp, while still detected in Sox9 positive cells (Sertoli) and interstitial cells (Additional file 3: Fig. S3A).

**Fig. 3:**
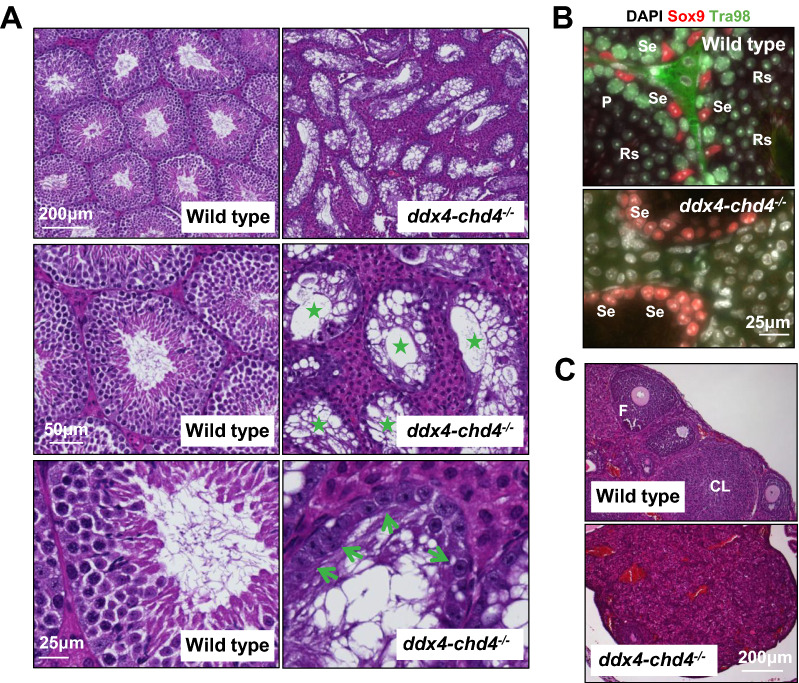
*Ddx4-chd4*^*−/−*^ mice show profound defects in gametogenesis. **A** H&E-stained histological sections of wild type and *ddx4-chd4*^*−/−*^ testis. Stars mark seminiferous tubules with absent germ cells. Note unchanged number and morphology of Sertoli cells (indicated by green arrows). **B** Histological sections of wild type and *ddx4-chd4*^*−/−*^ testis showing seminiferous tubules immunolabeled with Sox9 (a marker of Sertoli cells) and Tra98 (to mark germ cells). *P* pachytene cells, *Se* sertoli cells, *Rs* rounded spermatids. **C** H&E-stained histological sections of wild type and *ddx4-chd4*^*−/−*^ ovaries. *F* follicles, *CL* corpora lutea

We found that adult *ddx4-chd4*^*−/−*^ males develop testicular hypoplasia with hyperplasia of interstitial cells and lack spermatozoa (Fig. 3A). The number of seminiferous tubules is similar between wild type and mutant animals, but the diameter is reduced (wild type, mean ± SD, 287 ± 34, *n* = 400 seminiferous tubules cross sections (3 different mice, 2-month-old) versus *chd4*^*−/−*^ 148 ± 21.3, *n* = 210, *P* < 0.0001 *t* test).

Analysis of 2-month-old *ddx4-chd4*^*−/−*^ testes revealed a total loss of germ cells (marked by Tra98) in seminiferous tubules (Fig. 3B). No developing gametes were observed, including cell types at early stages (e.g., spermatogonia) (Fig. 3A, B). Sertoli cells develop normally in *ddx4-chd4*^*−/−*^ mice, consistent with the specific loss of Chd4 in germ cells at early stages of development. We did not observe differences in germ cell development between wild type and *ddx4-chd4*^*wt/−*^, and between wild type and *ddx4-chd3*^*−/−*^ mice (Additional file 4: Fig. S4A, B), consistent with their similar testis sizes (Fig. 2C, D).

We also analyzed H&E-stained histological sections of ovaries from 45-day-old wild type and *ddx4-chd4*^*−/−*^ female mice. We noted a significant reduction in ovary size, an increase in stromal cells, a reduced number of follicles (wild type, 10 ± 3, *n* = 6 mice versus *ddx4-chd4*^*−/−*^ 0.6 ± 0.9, *n* = 5, *P* < 0.0001 *t* test) and absent corpora lutea (wild type, 9 ± 1, *n* = 6 mice versus Ddx4-*Chd4*^*−/−*^ 0 ± 0, *n* = 5, *P* < 0.0001 *t* test) in the Ddx4-*Chd4*^*−/−*^ mice compared to wild type (Fig. 3C).

We conclude that deletion of *Chd3* has no apparent effect on gamete development. However, germ cell specific deletion of *Chd4* results in severe male and female germ cell developmental defects, possibly originated at premeiotic stages of development.

### Chd4 is required for neonate spermatogonia survival

**Fig. 4:**
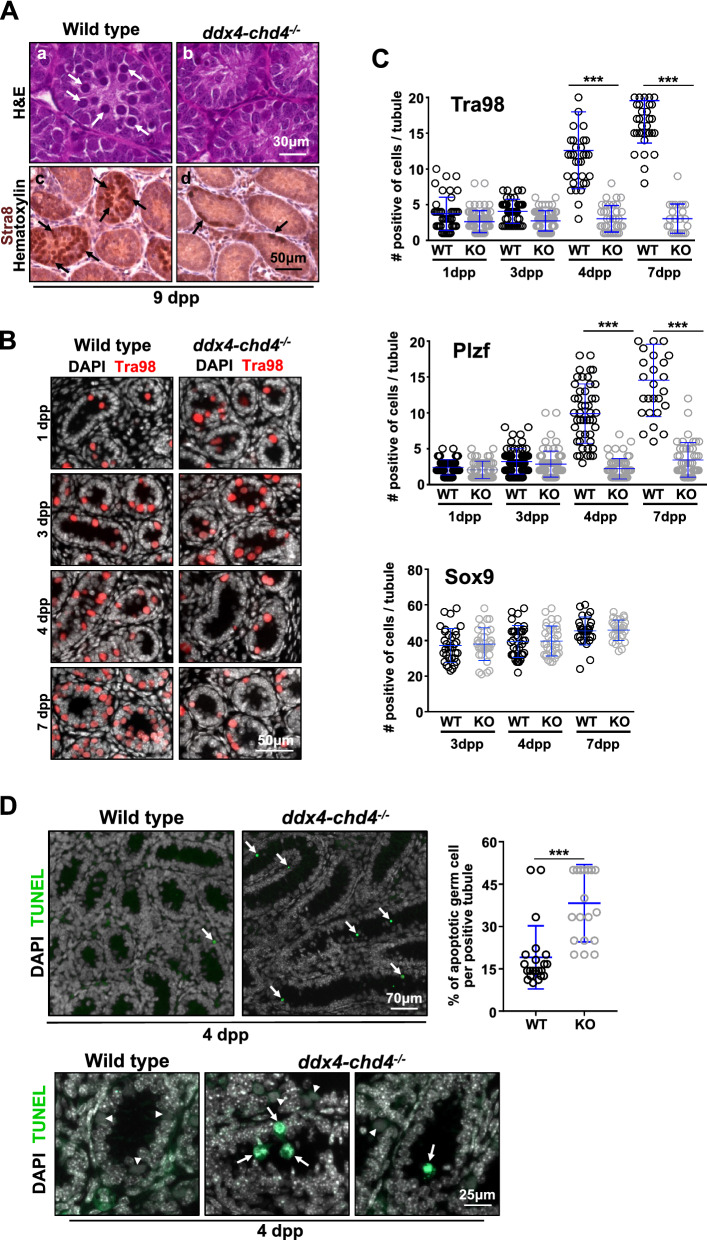
Spermatogonia cell survival requires Chd4. **A** Histological sections of wild type and *ddx4-chd4*^*−/−*^ testis cord from 9 days old mice stained with H&E (**a**, **b**) and Hematoxylin and immunostained with Stra8 antibodies (marking differentiating spermatogonia) (**c**, **d**). Arrows mark Stra8 positive cells present in wild type but reduced in number or absent in the mutant. Experiments were done in at least three different mice showing similar results. **B** Immunostaining of sections of developing testis reveals severe loss of germ cells (Tra98) in *chd4*^*−/−*^ testis cords. **C** Quantitation of number of cell positive for Tra98, Plzf, and Sox9. 4 dpp testis (Plzf, wild type, 9.9 ± 4.1, *n* = 47 seminiferous tubules; *chd4*^*−/−*^ 2.2 ± 1.4, *n* = 56, *P* < 0.0001, *t* test. Tra98, wild type 12.6 ± 5.3, *n* = 35; *chd4*^*−/−*^ 3.0 ± 1.9, *n* = 37, *P* < 0.0001, *t* test). 7 dpp testes (Plzf, wild type, 14.6 ± 5.0, *n* = 28; *chd4*^*−/−*^ 3.4 ± 2.4, *n* = 45, *P* < 0.0001, *t* test. Tra98, wild type 19.6 ± 5.9, *n* = 48; *chd4*^*−/−*^ 3.0 ± 2.1, *n* = 24, *P* < 0.0001, *t* test). Experiments were done in three different mice showing similar results combined in our quantification. **D** Higher cell death in *chd4*^*−/−*^ knockout testes than in wild-type controls at 4 days post-partum. Apoptotic cells were visualized using TUNEL staining in three mice of each genotype. Series of images (tiles) were used to construct the lower magnification (top panel) and the magnification showed a single tile in the bottom panel. The percentage of apoptotic cells was counted only in the tubule cross sections that contained apoptotic cells and was normalized to total number of germ cells. 19.05 ± 11.17, *n* = 468 (mean ± SD, *n* = number of seminiferous tubules counted) wild type and 38.25 ± 13.72 *chd4*^*−/−*^, *n* = 344; *P* < 0.0001 (two-tailed Student *t* test). The arrows indicate apoptotic cells and arrowhead non-apoptotic germ cells

The severe phenotypes observed in *ddx4-chd4*^*−/−*^ mice (Figs. 2 and 3) prompted us to investigate spermatogonial differentiation during testis development in newborns. Testis sections from 9 dpp *ddx4-chd4*^*−/−*^ mice stained with H&E showed a markedly reduced number of germ cells (Fig. 4A) as well as differences in cell composition, compared to those from age-matched wild-type mice. To analyze this in detail, we examined the presence of cells expressing Stra8 (Fig. 4A), which marks differentiating spermatogonia, Sycp3 and γH2AX which are markers of primary spermatocytes and Tra98, a marker for germ cells (Additional file 5: Fig. S5). Whereas tubules from 9 dpp wild-type mice contained cells expressing Tra98 (45 ± 10, *n* = 66 seminiferous tubules counted obtained from 3 mice) and Stra8 (18.8 ± 8.4, *n* = 36 obtained from 3 mice), tubules from *ddx4-chd4*^*−/−*^ mice showed a near absence of cells expressing these markers (Tra98 4.6 ± 3, *n* = 60 obtained from 3 mice, *P* < 0.0001, *t* test; Stra8 2.5 ± 3.5, *n* = 42 obtained from 3 mice, *P* < 0.0001, Student *t* test) (Fig. 4A). Testes sections from 9 dpp *ddx4-chd4*^*−/−*^ mice also showed a reduction in primary spermatocytes expressing the meiotic prophase I markers Sycp3 and γH2AX compared to those from 9 dpp wild-type mice (Additional file 5: Fig. S5A, B). Together, the results further suggest that testis defects in *ddx4-chd4*^*−/−*^ mice begin early, during pre-meiotic stages of postnatal development, leading to an absence of germ cells in adults.

To pinpoint when the testes defects originate in *ddx4-chd4*^*−/−*^ mice, we stained testes sections from 1 to 21 dpp mice for the expression of Tra98 (all germ cells). We observe that as general trend, in all analyzed stages, the number of Tra98 positive cells was reduced in *ddx4-chd4*^*−/−*^ mice compared to wild type, progressing to total absence of germ cells (Fig. 4B, C and Additional file 6: Fig. S6). Both Plzf-positive and Tra98-positive cells were substantially reduced in c*hd4*^−/−^ testis compared to wild-type testis at 4 dpp and 7 dpp (Fig. 4C). We observed equal numbers of Sox9-positive (Sertoli) cells in testes from wild type and *ddx4-chd4*^*−/−*^ mice at 3, 4, and 7 dpp (Fig. 4C), as expected for the specific loss of Chd4 in spermatogonia cells.

Given that Chd4 may act as a regulator of cell-cycle progression, we then examined whether the rapid loss of Plzf-positive neonate spermatogonia in *ddx4-chd4*^*−/−*^ testes was due to altered proliferative activity. We conducted EdU incorporation study to test this possibility. 4 dpp mice were injected with EdU and analyzed 3 h later, after which we assayed its incorporation in Plzf-positive spermatogonia in whole mounts of seminiferous tubules (Additional file 7: Fig. S7A). We found that spermatogonia cell proliferation (Plzf/EdU^+^ cells) in wild type and *ddx4-chd4*^*−/−*^ is proportionally the same (Additional file 7: Fig. S7C). In addition, reduced amount of total Plzf-positive cells was found in *ddx4-chd4*^*−/−*^ compared to wild type in the whole-mounting experiment (Additional file 7: Fig. S7B).

To determine whether cell death contributed to the loss of *ddx4-chd4*^*−/−*^ neonate spermatogonia (Fig. 4C), we performed TUNEL assay in paraffin embedded testis sections of wild type and *ddx4-chd4*^*−/−*^ 4 days old mice. At this age the testis is mostly constituted by spermatogonia and Sertoli cells, which can be easily distinguished by DAPI nuclear staining patterns. We found a significant increase in the percentage of apoptotic germ cells, but not Sertoli cells in *ddx4-chd4*^*−/−*^ testis compared to wild-type mice (Fig. 4D).

We conclude that the possible cause of spermatogonia failure in *ddx4-chd4*^*−/−*^ mice is in the survival/maintenance of neonate undifferentiated spermatogonia.

### Genome wide Chd4 chromatin binding

**Fig. 5:**
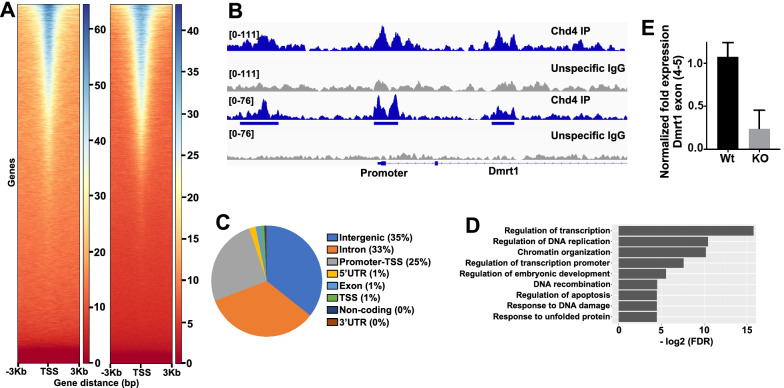
Chd4 genome-wide chromatin binding profile and analysis. **A** Heatmaps showing Chd4 occupancy centered in transcription start site (TSS) for all genes. The left and right panel correspond to heatmaps obtained from enriched spermatogonia fractions and whole 7 dpp testis, respectively. **B** Examples of Chd4 occupancy at or near *dmrt1* promoter assessed by ChIP-seq. The top and bottom panels correspond to profiles obtained from enriched spermatogonia fractions and whole 7 dpp testis, respectively. **C** Pie chart depicting genomic features associated with Chd4 peaks. **D** Gene ontology analysis of genes associated to Chd4 ChIP-seq signals. **E** RT-PCR analysis for *dmrt1* expression at exon 4–5 boundary in wild type and *chd4* knockout testis

To further investigate the mechanism of Chd4 requirement in neonate spermatogonia, we aimed to identify genes that directly interact with Chd4. As an approximation, we generated genome-wide chromatin-binding profiles of Chd4 by ChIP-seq in whole 7 dpp testis and in enriched fractions of spermatogonia from 7 dpp wild-type testes (Thy1.2+ and c-Kit+ cells) (Fig. 5). Heatmaps and direct visualization of profiles revealed a prominent enrichment of Chd4 in gene regulatory elements (Fig. 5A, B and Additional file 8: Table S1), suggesting that Chd4 regulates gene transcription in neonate spermatogonia. We observed a good correlation between both ChIP-seq profiles, with most peaks obtained from enriched fractions of spermatogonia from 7 dpp wild-type testes also present in chromatin-binding profiles from whole 7 dpp testis.

To gain greater insight into the genes potentially regulated by Chd4, we annotated shared peaks of Chd4 to the closest gene and performed functional analysis using DAVID platform [22]. Among the most significantly enriched terms obtained using Genome Ontology Biological Processes (GO-BP, FDR < 0.05) we found: regulation of transcription, regulation of DNA replication, chromatin organization/remodeling, and regulation of apoptotic processes (Additional file 9: Table S2). On an attempt to narrow down genes that could be directly regulated by Chd4, and that may explain the observed cellular phenotype, we search for genes related to stem cell survival/differentiation with a Chd4 peak near the promoter. We found Dmrt1, a known gene acting in spermatogonia cell maintenance/survival [23]. We noted other genes with recognized spermatogonia function, such as Foxo3, involved in cell self-renewal and differentiation [24]; Rest, involved in survival of PGCs [25]; and Mettl14, which inactivation causes depletion of SSCs possibly by dysregulation of transcripts required for spermatogonia proliferation/differentiation [26] (Additional file 10: Table S3).

### Chd4, Dmrt1, and Plzf work together in a regulatory axis involved in spermatogonia cell survival

**Fig. 6:**
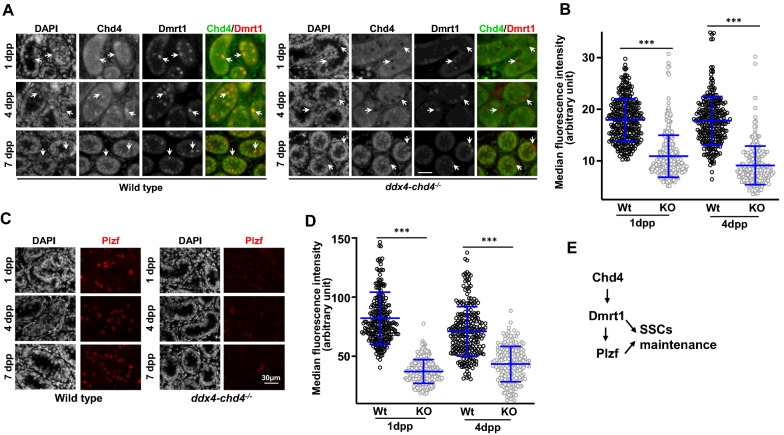
Effect of *chd4* deletion in Dmrt1 and Plzf expression. **A** Histological sections of wild type and ddx4-*chd4*^*−/−*^ testis from 1, 4, and 7 dpp mice stained with Chd4 and Dmrt1 antibodies. Arrows indicate examples of Chd4 positive spermatogonia (Wt) or Chd4 negative staining in ddx4-*chd4*^*−/−*^ spermatogonia and the correspondent cell stained for Dmrt1. **B** Quantitation of fluorescence intensity of Dmrt1 expression on spermatogonia is shown for wild type (Wt) and ddx4-*chd4*^*−/−*^ knockout at ages of 1 dpp (Wt = 82.17 ± 22.02, *n* = 272; and KO = 37.20 ± 10.01, *n* = 287, *P* < 0.0001, Student *t* test) and 4 dpp (Wt = 71.09 ± 20.93, *n* = 242; and KO = 43.47 ± 14.88, *n* = 198, *P* < 0.0001, Student *t* test). Values represent the median fluorescence intensity ± standard deviation, *n* = total number of cells analyzed from 3 biological replicates using 3 different mice. **C** Histological sections of wild type and ddx4-*chd4*^*−/−*^ testis from 1, 4, and 7 dpp mice stained with antibodies against Plzf. **D** Quantitation of fluorescence intensity of Plzf expression on spermatogonia is shown for wild type and ddx4-*chd4*^*−/−*^ knockout at ages of 1 dpp (wt = 17.94 ± 4.10, *n* = 300; and ddx4-*chd4*^*−/−*^  = 10.89 ± 4.07, *n* = 339, *P* < 0.0001, Student *t* test) and 4 dpp (Wt = 17.78 ± 4.70, *n* = 282; and ddx4-*chd4*^*−/−*^ = 9.11 ± 3.75, *n* = 230, *P* < 0.0001, Student *t* test). Values represent the median fluorescence intensity ± standard deviation, *n* = total number of cells analyzed from 3 biological replicates using 3 different mice. **E** Diagram representing Chd4, Dmrt1, and Plzf relationship in spermatogonia stem cell maintenance. Note that our studies do not address the possibility that Plzf may be a direct target of CHD4

Our results show that Chd4 is required for spermatogonia maintenance/survival. We then reason that Chd4 may interact with genes that have been described to work in spermatogonia maintenance. Indeed, Dmrt1 has been show to function in spermatogonia stem cells maintenance, and this function seems to be mediated by direct regulation of *plzf* expression, another transcription factor required for spermatogonia maintenance [23]. To test our hypothesis, we first performed *dmrt1* RT-PCR analysis in enriched fractions of spermatogonia using an exon 4–5 specific probe. We observed that *dmrt1* expression was substantially reduced in *chd4* knockout testis compared to wild type (Fig. 5E). We also immunostained paraffined testes sections from 1, 4, and 7 dpp mice for the presence of Dmrt1 (Fig. 6A, B). Compared to wild type, *ddx4-chd4*^*−/−*^ mutant showed a clear reduction in Dmrt1 immunosignal.

Recent work described that Dmrt1 controls *plzf* expression, which is a transcription factor required for spermatogonia maintenance [23]. We then tested the effect of *chd4* depletion in Plzf expression. Plzf immunostaining of testes sections from 1, 4, and 7 dpp mice revealed a significantly reduction of this protein in *chd4* knockout cells with respect to wild type (Fig. 6C, D). This is consistent with a model by which Chd4 control of *dmrt1* and its downstream targets influences cell survival/maintenance (Fig. 6E).

We concluded that Chd4 participates in the maintenance/survival of neonate spermatogonia stem cell possibly through transcriptional regulation of genes participating in these critical processes. We note, however, that the dramatic phenotype observed in *ddx4-chd4*^*−/−*^ spermatogonia likely reflect CHD4 targeting a wide spectrum of genes participating in different pathways.

## Discussion

In this work, we examined the potential function of two critical NURD catalytic subunits, Chd4 and Chd3, in spermatogenesis. Our data suggest that a Chd4-NURD (but not Chd3-NURD) complex controls neonate spermatogonia development at early stages of testis development. Germline deletion of *chd4*, but not *chd3*, results in a severe loss of germ cells specifically at early stages of the testis cord development. *Chd4* deletion affects spermatogonia, with the first obvious consequences in undifferentiated spermatogonia. Our results agree with a recent report, in which using an in vitro approach, knockdown of Chd4 in spermatogonia cultures followed by cell transplantation in cell-ablated recipient testis, resulted in reduced viability of undifferentiated spermatogonia [16].

Most CHDs are expressed in testis; however, their insertion into the NURD complex seems to be developmentally regulated, with apparent different patterns of expression during gametogenesis. Chd5 [8–10] has been shown to be expressed and required to compact chromatin in postmeiotic stages of spermatogenesis [13, 27]. Our results show that Chd4 is expressed and functions at premeiotic stages of gametogenesis.

The functions of Chd4-NURD in neonate spermatogonia development and male gametogenesis are further revealed by our ChIP-seq (Fig. 5) and cytological analysis showing that Chd4 binds to the promoter of *dmrt1* and regulates its expression (Figs. 5 and 6). Dmrt1 function in maintenance of spermatogonia stem cells has been proposed to be mediated by direct regulation of Plzf gene expression, another transcription factor required for spermatogonia maintenance [23]. In agreement with this possibility, we observed that the amount of Plzf was significantly reduced in *chd4* knockout versus wild-type cells. In sum, our work provides evidence of a regulatory axis in which Chd4 may control important genes involved in spermatogonia stem cell maintenance and survival. The effect we observed with in vivo Chd4 deletion on *dmrt1* expression in spermatogonia (and possibly downstream genes, such as *sohlh1*) is in contrast with a recent report showing an increase in *dmrt1* and *sohlh1* expression measured by single cell RNA-seq in *chd4* siRNA knockdown cultured spermatogonia cells [16]. These contrasting results, and some of their implications, such as on the primary function of Chd4 as an activator of genes involved in spermatogonia survival, may be assigned to differences in the experimental system or experimental approaches utilized. Additional work will be required to test *sohlh1* expression level in Chd4 knockout spermatogonia, another *dmrt1* direct target involved in cell survival and differentiation [23, 28, 29], as well as in the *stra8* gene, the latter which precocious expression is detected in *dmrt1* knockout mice. We note that the dramatic cellular phenotype we observed after Chd4 depletion in spermatogonia may be more accurately explained by Chd4 activity targeting several genes and different pathways.

## Materials and methods

### Mice

*Chd4*-floxed mice (*chd4*^*fl/fl*^*)* have been described [30]. *chd3-*floxed mice were generated by Cyagen Biosciences using homologous recombination of a targeting vector in C57Bl/6 embryonic stem cells. The targeting vector incorporated a 5′ LoxP site inserted between exons 12 and 13 and a 3′ LoxP site inserted between exons 20 and 21 of the wild-type *chd3* allele. Transgenic *Cre* recombinase mice *ddx4-Cre*^FVB−Tg(*ddx4*−cre)1Dcas/J^ was purchased from The Jackson Laboratory (Bar Harbor, ME). *chd3* or *chd4* gonad-specific knockouts and wild-type heterozygotes littermates were obtained from crosses between female homozygous *flox/flox* mice with male heterozygous Cre/+; *chd3* wt/flox and/or *chd4* wt/flox mice.

All experiments conformed to relevant regulatory standards guidelines and were approved by the Oklahoma Medical Research Foundation-IACUC (Institutional Animal Care and Use Committee).

### Mice genotyping

Characterization of wild type and *floxed* alleles was carried out by PCR using the following oligonucletides: *chd3* forward 5′-GGGTGGAGGTGGAAAGTGTA, *chd3* reverse 5′-AGAGGACAGGTCACAGGACAA, *chd4* forward 5′-TCCAGAAGAAGACGGCAGAT and *chd4* reverse 5′-CTGGTCATAGGGCAGGTCTC. The presence of *cre* recombinase allele were determine by PCR using the following primers: *ddx4*-Cre forward 5′-CACGTGCAGCCGTTTAAGCCGCGT, *ddx4*-Cre reverse 5′-TTCCCATTCTAAACAACACCCTGAA.

### Real-time PCR

Total RNA was isolated from adult testis or from enriched fractions of spermatogonia with the Direct-zol RNA MiniPrep Plus kit (Zymo Research). RNA (2.0 μg) was oligo-dT primed and reverse-transcribed with the high-capacity RNA-to-cDNA kit (Applied Biosystems). Exon boundaries of *chd4 and chd3* were amplified using TaqMan Assays (Applied Biosystems) as directed by the manufacturer using Beta-2 macroglobulin as standard. TaqMan Mm01190896_m1 (*chd4*), Mm01332658_m1 (*chd3*), Mm00437762_m1 (Beta-2 microglobulin), and Mm00443809_m1 (*dmrt1*). Gene expression was normalized with respect to wild type with wild-type expression levels considered to be 1.

### Western blot cell lysates

Total testis or enriched cells fractions were lysed in ice-cold protein extraction buffer containing 0.1% Nonidet P-40, 50 mM Tris–HCl, pH 7.9, 150 mM NaCl, 3 mM MgCl_2_, 3 mM EDTA, 10% glycerol, 1 mM DTT, 1 mM PMSF and protease inhibitors (ThermoFisher Scientific, A32965) followed by sonication (3 pulses of 10 s) using micro ultrasonic cell disrupter (Kontes). The relative amount of protein was determined measuring absorbance at 260 nm using NanoDrop 2000c spectrophotometer (ThermoFisher Scientific). Proteins were solubilized with 2× sample buffer (4% SDS, 160 mM Tris–HCl, pH 6.8, 20% glycerol, 4% mM β-mercaptoethanol, and 0.005% bromophenol blue) and 30 µg/lane of sample were separated by 4–15% gradient Tris–acetate SDS–PAGE and electro transferred to PVDF membrane (Santa Cruz Biotechnology, sc-3723). The blots were probed with individual primary antibodies, and then incubated with HRP-conjugated goat anti-mouse or rabbit antibody as required. In all blots, proteins were visualized by enhanced chemiluminescence, and images acquired using Western Blot Imaging System c600 (Azure Biosystems). ImageJ software were used for quantification of non-saturated bands and α-tubulin were used for normalization. Antibodies used are detailed in Additional file 11: Table S4.

### Histology and immunostaining

Testes and ovaries were dissected, fixed in 10% neutral-buffered formalin (Sigma) and processed for paraffin embedding. After sectioning (5–8 µm), tissues were positioned on microscope slides and analyzed using hematoxylin and eosin using standard protocols. For immunostaining analysis, tissue sections were deparaffinized, rehydrated and antigen was recovered in sodium citrate buffer (10 mM Sodium citrate, 0.05% Tween 20, pH 6.0) by heat/pressure-induced epitope retrieval. Incubations with primary antibodies were carried out for 12 h at 4 °C in PBS/BSA 3%. Primary antibodies used in this study are detailed in Additional file 11: Table S4, following three washes in 1× PBS, slides were incubated for 1 h at room temperature with secondary antibodies. A combination of fluorescein isothiocyanate (FITC)-conjugated goat anti-rabbit IgG (Jackson laboratories) with Rhodamine-conjugated goat anti-mouse IgG and Cy5-conjugated goat anti-human IgG each diluted 1:450 were used for simultaneous triple immunolabeling. Slides were subsequently counterstained for 3 min with 2 µg/ml DAPI containing Vectashield mounting solution (Vector Laboratories) and sealed with nail varnish. We use Zen Blue (Carl Zeiss, inc.) for imaging acquisition and processing.

### Enrichment of spermatogonia populations

Our procedure of cell enrichment followed [31, 32]. Briefly, testis from 7 dpp mice (or any other indicated age) were removed from mice and placed in a Petri dish containing Dulbecco’s Modified Eagle Medium (DMEM without phenol red). After detachment of the tunica albuginea, the seminiferous tubules were loosen using forceps and incubated in a 15 mL tube containing DMEM containing 1 mg/mL of collagenase, 300 U/mL of hyaluronidase and 5 mg/mL DNAse I (StemCell Technologies) under gentile agitation for 10 min. The seminiferous tubule clumps were pelleted by gravity and the cell suspension containing interstitial cells was discarded. The tubules were then incubated of with 0.05% Trypsin–EDTA solution (Mediatech Inc) for 5 min and the reaction was stopped by adding 10% volume of 10% BSA in PBS. Single cell suspension was obtained by mechanical resuspension followed by filtration through a 40-µm-pore-size cell strainer and dead cells were removed using Dead Cell Removal Kit (Miltenyi Biotec 130-090-101). Differentiating c-KIT+ neonate spermatogonia cells were magnetically labeled with CD117 (c-KIT+) MicroBeads (Miltenyi Biotec 130-091-224) and isolated using MS columns (Miltenyi Biotec 130-042-201) according to manufacturer’s instructions. After the depletion of the c-KIT+ cells, the population of undifferentiated neonate spermatogonia cells were separated using CD90.2 (THY1.2+) MicroBeads (Miltenyi Biotec 130-121-278). Relative enrichment of cell populations was evaluated by STRA8 (c-Kit fractions) or PLZF (THY1.2 fractions) western blots (Fig. 1B). After c-kit and THY1.2 separation, the flow-through mostly contained Sertoli cells (SOX9 positive, Additional file 3: Fig. S3). The number of cells obtained from a pool of 4 mice testis at 7 dpp was approximately 3.43 × 10^5^ in THY1.2 fractions and 5.71 × 10^5^ in c-Kit fractions.

### Primary spermatocyte enrichment

Synchronized pachytene spermatocytes from the first spermatogenic wave were purified as described in [33]. Briefly, 2 dpp mice were injected for 7 consecutive days with WIN 18,446 to arrest germ cells as spermatogonia. The next day (9 dpp), mice were injected with retinoic acid (RA) to induce their coordinated maturation. 2 Mice were killed at 13 days after RA injection (22 dpp). Testes were disaggregated using proteases and meiocytes were then purified by fluorescence activated cell sorting (FACS). Unlike Romer et al., after testes disaggregation and prior to FACS, cells were not washed (by centrifugation and resuspension) to avoid breakage of fragile pachytene cells. Instead, cells were sorted from the protease-containing buffer. Purity and stage of cells was assessed by immunofluorescence of chromosome spreads using anti SYCP1 and anti SYCP3 antibodies. More than 80% of cells were at pachytene stage.

### Immunoprecipitation

Co-immunoprecipitation experiments were performed using testis of wild type or *ddx4*-*chd3*^*−/−*^ mouse (adult—2 months). After detunication, seminiferous tubules were loosen using forceps, washed twice with cold 1× PBS and lysed using ice-cold RIPA buffer (50 mM Tris pH 7.5, 150 mM NaCl, 1% NP40, 0.5% Deoxycholate) containing protease inhibitors (ThermoFisher Scientific, A32965), sheared using 23 G needle, incubated on ice for 15 min and centrifugated at 1000×*g* for 10 min at 4 °C. Supernatant were collected in a separate tube, the pellet was resuspended in RIPA buffer, disrupted by sonication (3 pulses of 10 s) and centrifuged 12,000×*g*. This second supernatant was combined with the previous one and protein concentration was determined. We used 1 mg of protein for each immunoprecipitation. Lysates were pre-cleared with protein G magnetic beads (BioRad, 161-4023) for 1 h at room temperature and incubated with rabbit anti-Chd3 (5 μg, Bethyl A301-220A), rabbit anti-Chd4 (2 μg, Abcam ab72418), or rabbit IgG (5 μg Jackson ImmunoResearch, 011-000-003). Lysates were rotated overnight at 4 °C and immune complexes were collected with protein G magnetic beads (2 h at 4 °C). Beads were washed 4 times with washing buffer (50 mM Tris pH 7.5, 150 mM NaCl, 0.1% TX100, 5% glycerol) and two times with PBS. Proteins were eluted by boiling the beads with 2× sample buffer and analyzed by SDS–PAGE as described above.

### EdU-based proliferation assay

Mice at indicated age received subcutaneous injection of EdU (50 mg/kg) (Invitrogen, A10044) 3 h prior euthanasia. After that, testes were removed and processed for whole-mount immunohistochemistry. EdU was detected by incubation of testis samples with reaction mix (2 mM CuSO_4_, 50 mM ascorbic acid and 2 mM Alexa Azide conjugates (488 or 647) in PBS) for 3 h at room temperature.

### Whole-mount seminiferous tubules

Immunohistochemistry of whole-mount seminiferous tubules was performed as described [34]. Briefly, after detachment of the tunica albuginea, the seminiferous tubules were loosen using forceps and incubated in a 15 mL tube containing DMEM containing 1 mg/mL of collagenase, 300 U/mL of hyaluronidase and 5 mg/mL DNAse I (StemCell Technologies) under gentile agitation for 10 min. The seminiferous tubules clumps were pelleted by gravity and the cell suspension containing interstitial cells was discarded. Seminiferous tubules were fixed for 4 h in 4% PFA (pH7.2 in PBS) at 4 °C. After extensively wash in PBS, the tubules were permeabilized with series of MeOH/PBS (25, 50, 75, 95%, and twice in 100% MeOH) for 15 min at room temperature, treated with MeOH: DMSO: H_2_O_2_ (4:1:1), and rehydrated with MeOH/PBS (50, 25% and twice in PBS). Samples were incubated in ice-cold blocking solution PBSMT (PBS with 2% non-fat dry milk and 0.5% triton X-100) for 3 h and then over-night at 4 °C with indicated primary antibodies under gentle rotation. Seminiferous tubules were washed in PBSMT (5 × 1 h) and incubated with dye conjugated (Alexa488 or TRITC) goat anti-mouse or rabbit antibody as required. The tubules were mounted in raised coverslips glass slides.

### ChIP-seq

We used whole 7 dpp testes or undifferentiated and differentiating spermatogonia cell fractions obtained by affinity purification (C-kit and Thy1, respectively) of total wild-type testes. ChIP was performed as previously described with modifications [35]. Cells were fixed for 5 min 1% methanol-free formaldehyde, quenched for 5 min with 125 mM glycine, washed twice with PBS 1× and suspended in 5 mL of PBS 1× containing 1 mM of PMSF. Cells were dounced 20 strokes with a ‘B’ pestle, and then, pelleted at 2000 rpm for 5 min at 4 °C. The cells were suspended in in 5 mL of Lysis buffer 1 containing 1 mM PMSF (10 mM Tris–HCl pH8, 10 mM EDTA, 0.5 mM EGTA, 0.25% Triton X-100) and centrifuged at 2000 rpm for 5 min at 4 °C. The same procedure was repeated with 5 mL of Lysis buffer 2 containing 1 mM PMSF (10 mM Tris–HCl pH8, 1 mM EDTA, 0.5 mM EGTA, 200 mM NaCl). The cells were finally resuspended in 1 mL of ChIP buffer containing 1X protease inhibitor cocktail, Roche (50 mM Tris–HCl pH8, 1 mM EDTA, 150 mM NaCl, 1% Triton X-100, 0.1% Na deoxycholate, 0.1% SDS) and sonicated for 15 or 24 min for spermatogonia or whole testes, respectively, using the Covaris E220 evolution (peak power 140, duty factor 5, 200 cycles per burst). The sample was cleared at 12,000×*g* for 10 min at 4 °C, and the chromatin was incubated with anti-Chd4 antibody (ab 70469, Abcam) overnight at 4 °C. Antibody/chromatin complexes were captured with ChIP-grade protein A/G magnetic beads (ThermoFisher) for 2 h at 4 °C and washed 2 times with increasing salt concentration (20 mM Tris–HCl pH8, 2 mM EDTA, 150–500 mM NaCl, 1% Triton X-100, 0.1% Na deoxycholate) and once with a lithium buffer (10 mM Tris–HCl pH8, 1 mM EDTA, 250 mM LiCl, 1% Igepal, 0.7% Na deoxycholate). The beads were washed twice with TE buffer pH7.4 (10 mM Tris–HCl, 1 mM EDTA) and DNA was eluted at 65 °C with agitation for 30 min using 150 ~ µL 1% SDS + 100 mM NaHCO_3_ made freshly (twice). Cross-links were reversed overnight by adding 5 µL of 5 M NaCl and incubating overnight at 65 °C. DNA was treated with 3 µL RNaseA (Qiagen cat # 19,101) for 30 min at 37 °C and then with 5 µL of proteinase K (approximately 3 U, Qiagen, cat. # 19131) for 1 h at 56 °C. The DNA was purified using the miniElute PCR purification kit using 7 volumes of PB buffer, washing with ethanol twice and eluting twice in 12 µL (Qiagen, cat. # 28004) and quantified using Qubit (Life Technologies) before library.

### Library preparation and sequencing

Spermatogonia-purified ChIP and its input libraries were prepared using an in-house method (see below), and sequenced single read 85 bp on an Illumina NextSeq 500 instrument. Whole testes ChIP and its input libraries were prepared using ACCEL-NGS® 1S PLUS DNA LIBRARY KIT (cat. # 10024) in conjunction with Swift unique dual indexing kit (cat. # X9096), following manufacturer’s instructions, and sequenced as paired end 150 bp on an Illumina Novaseq 6000 instrument. Both sequencings were done at OMRF Clinical Genomics Core.

Before sequencing, samples were quantified by qPCR using Kapa library quantification kit (cat # KK4854), and size and quality of DNA were assessed using Agilent Tape station.

#### In-house library preparation method

20 µL of the eluted DNA was incubated with 30 µL of end-repair mix (0.66 mM dNTP mix (NEB, cat. # N0447S), 100 U/mL T4 DNA polymerase (NEB, cat. # M0203L), 33 U/mL Klenow fragment (NEB, cat. # M0210S), 333 U/mL T4 PNK (cat. # M0201L), and 1.67× T4 PNK buffer) at 20 °C for 30 min. DNA was purified using Qiagen Minelute kit using 7 volumes of PB buffer and eluted in 12 µL of EB buffer. 10 µL of eluted DNA were mixed with 40 µL of A-tailing mix (0.25 mM dATP, 125 U/mL Klenow fragment (cat. # NEB M02105S), and Klenow buffer 1.25×) and incubated at 37 °C for 30 min. DNA was purified again using Qiagen Minelute kit using 7 volumes of PB buffer and eluted in 12 µL of EB buffer. Truseq single index adaptors (Illumina, cat. # 20015960 or 20015961) were diluted according to the DNA concentration (insert:adaptor molar ratio of 1:2, with maximal dilution of 1:50) and 1 µL of a specific diluted adaptor was added to each sample. 20 µL of ligation mix (30 U/µL T4 DNA ligase (cat. # NEB M0202L), and 1.58× ligase buffer) was added to 10 µL of the insert:adaptor mix and incubated for 30 min at 20 °C. DNA was purified using Qiagen Minelute kit using 7 volumes of PB buffer and eluted in 12 µL of EB buffer. Finally, DNA was amplified using the Kapa HiFi Hot Start library amplification kit (cat. # KK2621) according to manufacturer’s instructions.

### ChIP-seq data processing and analysis

#### Alignment and quality filtering

For samples prepared using adaptase technology (whole testes and its input), reads 1 and 2 were trimmed 10 bases at the beginning using fastp [36] (version 0.23.2), as recommended by the library preparation kit manufacturer. Further processing was done in parallel for all samples. Reads were adapter-trimmed and quality-pruned using fastp with default settings. Then, reads were aligned to mm10 genome using BWA–MEM [37] (version 0.7.15) with default settings except for option ‘-M’ for Picard compatibility. Picard (version 2.21.2, http://broadinstitute.github.io/picard/) and SAMtools (version 1.11) [38] were used to obtain mapping quality metrics, remove duplicates and filter reads. Only primary alignment reads that were not duplicated, properly paired, with a MAPQ > 30, and not placed in mitochondrial or unplaced-chromosomes were kept.

#### Greylist regions

Greylist regions were prepared for each input using the GreyListChIP R-package (R package version 1.24.0, https://bioconductor.org/packages/release/bioc/html/GreyListChIP.html) together with the Bsgenome.Mmusculus.UCSC.mm10 package (https://bioconductor.org/packages/release/data/annotation/html/BSgenome.Mmusculus.UCSC.mm10.html) using the following parameters: reps = 10, sampleSize = 10,000, *p* = 0.9999. These lists were merged and to mouse blacklist to create a single black-greylist, which was later used to filter peaks.

#### Peak calling

Peaks were called using MACS2 [39] (version 2.2.7.1) using broad mode and default values for the rest of the parameters.

#### Peak annotation and gene ontology analysis

Common peaks to both ChIPs were filtered by black-greylist and then annotated using HOMER’s annotatePeaks.pl script (22, version 4.10). To assign a score to the common peaks, we averaged the −log(*q* value) for both peaks. In cases in which a peak from one experiment was intersected by more than one peak from the other experiment, the −log(*q* values) from the same experiment were averaged first. Annotated peaks can be found at Additional file 8: Table S1. Functional annotation analysis was done using DAVID with default parameters and the list of annotated genes in which the promoter was closer than 5000 bp. Further processing and plotting were done using R (https://www.r-project.org/, version 4.1.1) and packages within tidyverse (https://joss.theoj.org/papers/10.21105/joss.01686). Functional analysis results can be found at Additional file 12: Table S5.

#### Heatmaps and IGV snapshots

Bigwig coverage tracks were generated using bamCoverage tool from deepTools (version 3.4.3, [40]) with a bin size of 10 bp, 40 bp smoothing and RPKM normalization. Average aggregate profiles and heatmaps were plot using deepTools as well (computeMatrix and plotHeatmap). Mapping statistics can be found in Additional file 12: Table S5.

### Statistical analyses

Results are presented as mean ± standard deviation (SD). Statistical analysis was performed using Prism Graph statistical software. Two-tailed unpaired Student’s *t* test was used for comparisons between 2 groups. *P* < 0.05 was considered statistically significant.

## Supplementary Information

**Additional file 1: Figure S1.** Chd4 expression during gametogenesis. Expression of Chd4 monitored by immunofluorescence in paraffin embedded testis sections of 1, 3, 4, 7, 9, 14, and 120 dpp mice. Spermatogonia cells are positive for Plzf and Sertoli cells are positive for Sox9.

**Additional file 2: Figure S2.** Spermatogonia enrichment. Analysis of Thy1+ and c-Kit+ fractions. Cells were attached to coverslips and immunostained with the indicated antibodies. **A** Quantification of germ cell shows that spermatogonia represents 61.5% (± 3.7%) from Thy1+ fractions (Plzf positive cells) and 65.4% (± 11%) from c-Kit fractions (Ddx4 positive cells). **B** Flow-through fraction obtained after use of the Thy1 and c-Kit columns is composed of 60.6% ± 3.6% Sertoli cells. A minor fraction of Sertoli cells was also detected in Thy1+ (28.1 ± 6.3%) and c-Kit+ (25.4% ± 7.4%) fractions. Numbers represent average ± standard deviation from 2 biological replicates and 4 technical replicates.

**Additional file 3: Figure S3.** Specificity of Chd4 immunostaining and formation of Chd4- and Chd3-NURD complexes in developing gametes. **A** Antibodies against Chd4 colocalize with Plzf positive cells in seminiferous tubules of 1 dpp wild-type mice but no signal of Chd4 is detected in *ddx4-chd4*^*−/−*^ mice. Note Sertoli cells (Sox9) exhibit Chd4 immunosignal in both wild type and *ddx4-chd4*^*−/−*^ mice. This is a representative image out of three different experiments using one wild type or *ddx4-chd4*^*−/−*^ per experiment. Note that the red channel (Plzf staining) in the image corresponding to *ddx4-chd4*^*−/−*^ correspond to a longer exposure to allow comparison to wild type. **B** Chd4 co-immunprecipitates Hdac2a, a core component of the NURD complex, independent of Chd3. Note the absence of Chd3 immunosignal in *ddx4-chd3*^*−/−*^ mice testis lysates (input). The asterisk indicates unspecific signal in the Chd3 blot and arrow indicates the specific band.

**Additional file 4: Figure S4.** Ablation of both *chd4* alleles are required for a Sertoli-only phenotype and Chd3 is dispensable for gametogenesis. **A** Histological sections of 2 months of old mice testis stained with H&E shows no differences between homozygous (*chd4*^*wt/wt*^) and the heterozygous (*ddx4-chd4*^*wt/−*^) mice controls, while *ddx4-chd4*^*−/−*^ mouse results in absence of germ cells. **B** H&E stained histological sections of wild type and *ddx4-chd3*^*−/*−^ testis. No differences in type or number of germ cell at any stage of development are observed between wild type and ddx4-*chd3*^*−/−*^ knockouts.

**Additional file 5: Figure S5.** Chd4 deletion results in deficient spermatogonia cell development and near absence of spermatocytes. **A** Histological sections of 9 dpp wild type and *ddx4-chd4*^*−/−*^ testis showing seminiferous tubules immunolabeled with Tra98 (a marker of germ cells) and Sycp3 and γH2AX (markers of primary spermatocytes). Arrows indicate examples of positive cells. **B** Quantitation of cell number per positive tubule shown in A. Tra98 positive cells in wild type (45 ± 10, *n* = 66) and *ddx4-chd4*^*−/−*^ (4.6 ± 3, *n* = 60, *P* < 0.0001, Student *t* test) mice. Sycp3 positive cells in wild type (25 ± 5, *n* = 45) and Ddx4-*Chd4*^*−/*−^ (2 ± 1, *n* = 45, *P* < 0.0001, Student *t* test) mice. γH2AX positive cells in wild type (20 ± 7, *n* = 38) and *ddx4-chd4*^*−/−*^ (2 ± 1, *n* = 38, *P* < 0.0001, Student *t* test) mice.

**Additional file 6: Figure S6.** Deficient spermatogonia cell survival and differentiation in *chd4*^*−/−*^ mice. Histological sections of wild type and *ddx4-chd4*^*−/−*^ testis cords from 1, 3, 4, 7, 9, 14, and 21 dpp mice stained with Tra98 antibodies. See quantification in Fig. 4C.

**Additional file 7: Figure S7.** Deficient spermatogonia cell survival in *chd4*^*−/*−^ knockout mice. **A** Immunostaining of whole mount seminiferous tubules reveals loss of spermatogonia (Plzf) in 4 dpp *ddx4-chd4*^*−/−*^ mice. EdU was used to mark proliferating cells. **B** Quantitation of number of Plzf positive cells per mm of seminiferous tubules [wild type (116 ± 48) and *ddx4-chd4*^*−/−*^ (54 ± 22), *P* < 0.0011, student *t* test]. Number of cells corrected by total length of seminiferous tubule analyzed in wild type versus *chd4*^*−/*−^ mutants. A total of 7.36 mm (833 cells, wild type) and 10.15 mm (478 cells, *ddx4-chd4*^*−/−*^) seminiferous tubule length were counted using four different mice. **C** Percentage of proliferative spermatogonia cells (Plzf^+^/EdU^+^) in wild type and *ddx4-chd4*^*−/−*^ (wild type (63% ± 13%) and *ddx4-chd4*^*−/−*^ (56% ± 16), *P* < 0.2816, Student *t* test).

**Additional file 8: Table S1.** List of Chd4 ChIP-seq signal.

**Additional file 9: Table S2.** Gene ontology analysis.

**Additional file 10: Table S3.** List of annotated Chd4-bounded genes possibly implicated in stem cell survival and/differentiation.

**Additional file 11: Table S4.** List of antibodies used in this work.

**Additional file 12: Table S5.** Mapping statistics corresponding to Chd4 ChIP-seq.

## Data Availability

All data generated or analyzed during this study are included in this published article and its Additional files.
