# Supplementary figures and images for: Mouse Chd4-NURD is required for neonatal spermatogonia survival and normal gonad development
Source: Epigenetics Chromatin. 2022 May 14;15:16. doi: 10.1186/s13072-022-00448-5 (PMC9107693; doi:10.1186/s13072-022-00448-5)

Figure S1

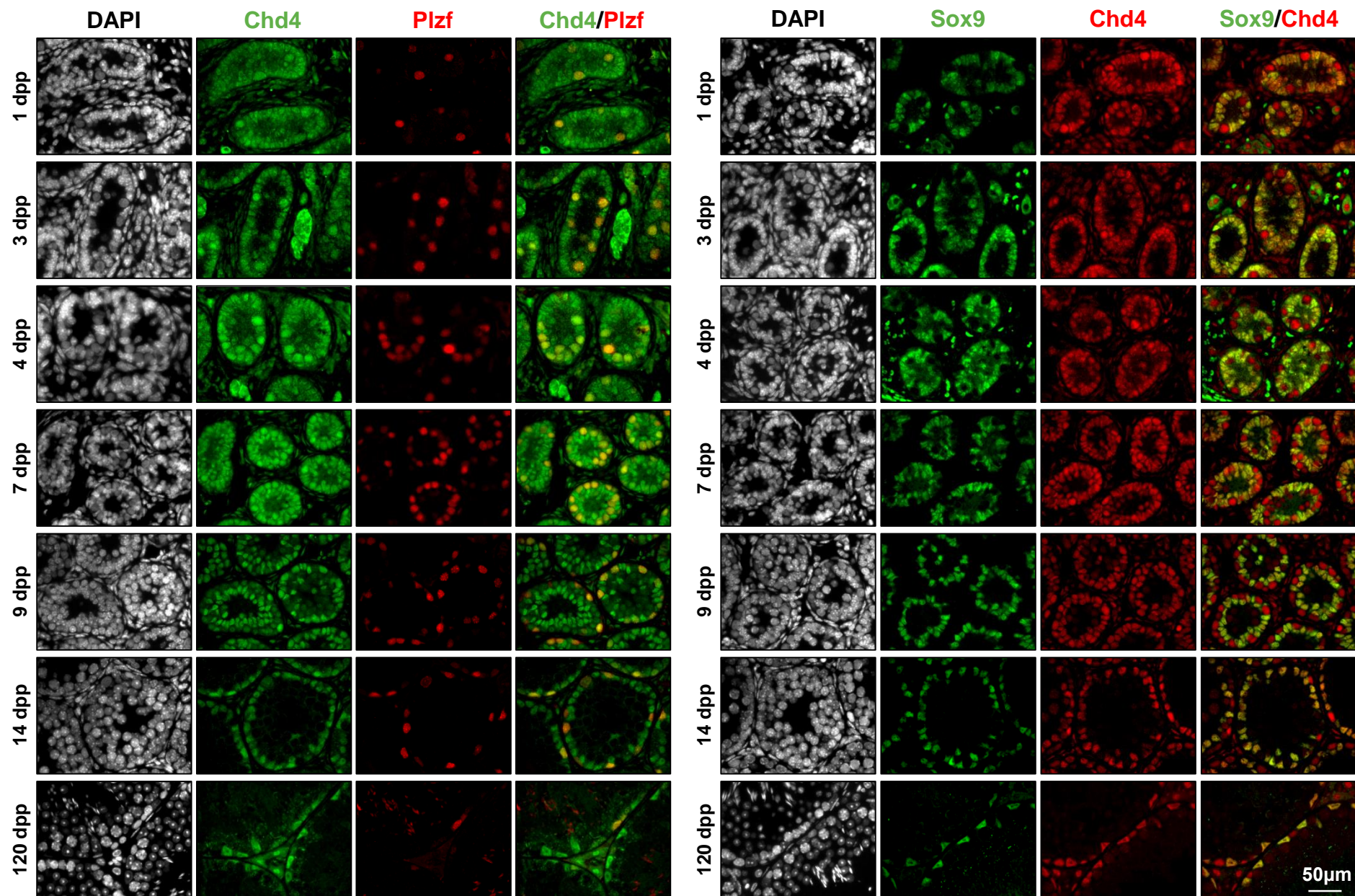

Supplement: Supplementary file 1 — Additional file 1: Figure S1. Chd4 expression during gametogenesis. Expression of Chd4 monitored by immunofluorescence in paraffin embedded testis sections of 1, 3, 4, 7, 9, 14, and 120 dpp mice. Spermatogonia cells are positive for Plzf and Sertoli cells are positive for Sox9. [file 13072_2022_448_MOESM1_ESM.pdf]

Figure S2

**A**

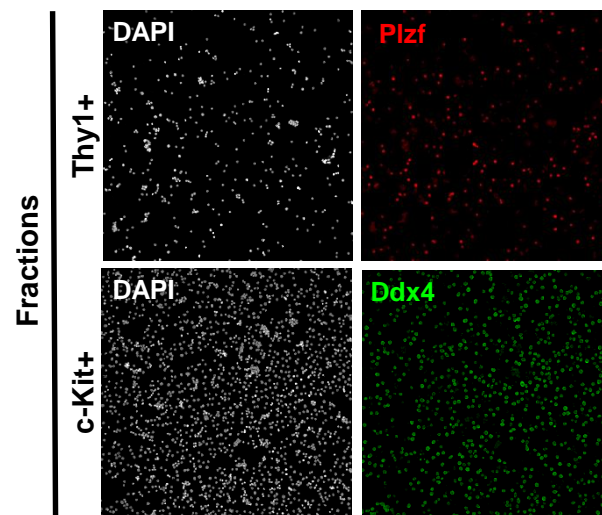

Enrichment of spermatogonia cell population

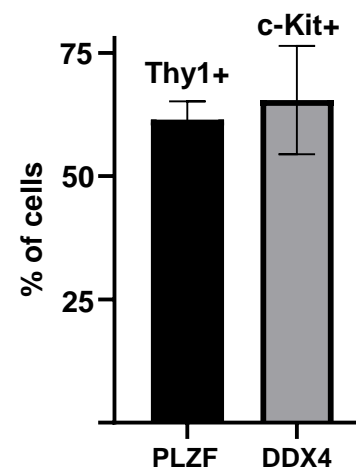

**B**

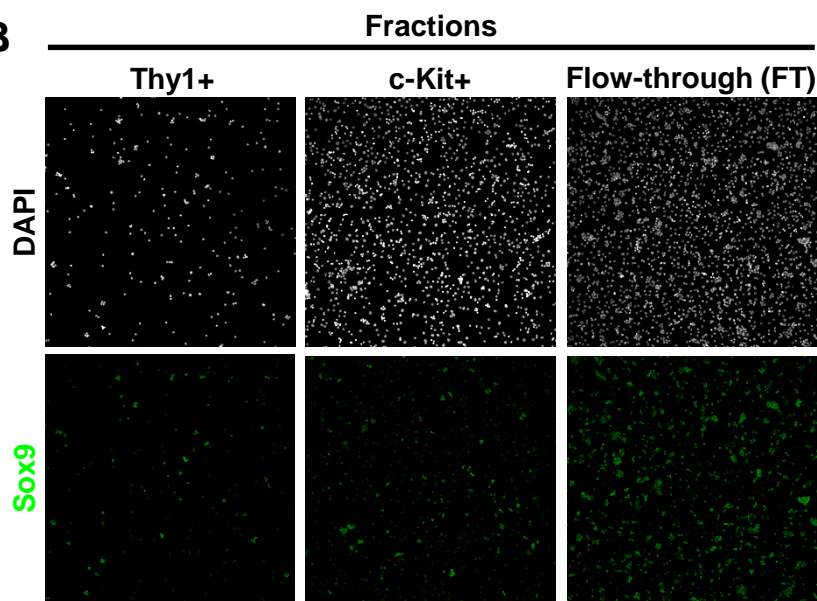

Presence of Sertoli cells

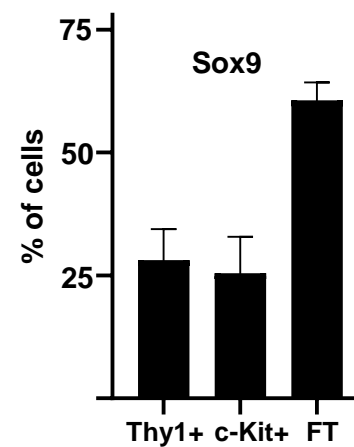

Supplement: Supplementary file 2 — Additional file 2: Figure S2. Spermatogonia enrichment. Analysis of Thy1+ and c-Kit+ fractions. Cells were attached to coverslips and immunostained with the indicated antibodies. A Quantification of germ cell shows that spermatogonia represents 61.5% (± 3.7%) from Thy1+ fractions (Plzf positive cells) and 65.4% (± 11%) from c-Kit fractions (Ddx4 positive cells). B Flow-through fraction obtained after use of the Thy1 and c-Kit columns is composed of 60.6% ± 3.6% Sertoli cells. A minor fraction of Sertoli cells was also detected in Thy1+ (28.1 ± 6.3%) and c-Kit+ (25.4% ± 7.4%) fractions. Numbers represent average ± standard deviation from 2 biological replicates and 4 technical replicates. [file 13072_2022_448_MOESM2_ESM.pdf]

**A**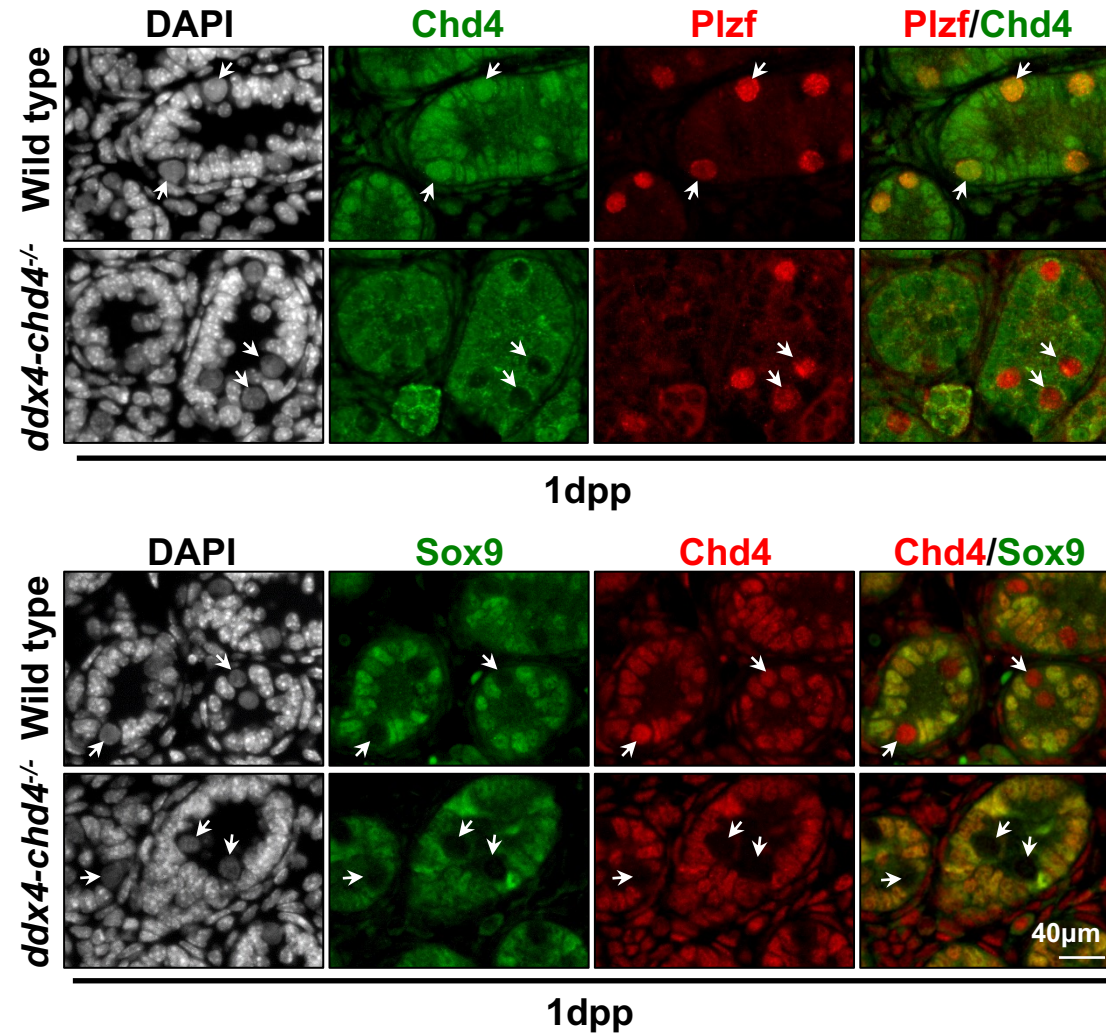

Figure S3

**B**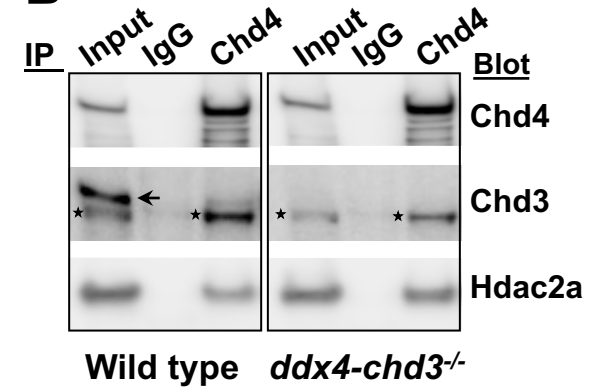

Supplement: Supplementary file 3 — Additional file 3: Figure S3. Specificity of Chd4 immunostaining and formation of Chd4- and Chd3-NURD complexes in developing gametes. A Antibodies against Chd4 colocalize with Plzf positive cells in seminiferous tubules of 1 dpp wild-type mice but no signal of Chd4 is detected in ddx4-chd4−/− mice. Note Sertoli cells (Sox9) exhibit Chd4 immunosignal in both wild type and ddx4-chd4−/− mice. This is a representative image out of three different experiments using one wild type or ddx4-chd4−/− per experiment. Note that the red channel (Plzf staining) in the image corresponding to ddx4-chd4−/− correspond to a longer exposure to allow comparison to wild type. B Chd4 co-immunprecipitates Hdac2a, a core component of the NURD complex, independent of Chd3. Note the absence of Chd3 immunosignal in ddx4-chd3−/− mice testis lysates (input). The asterisk indicates unspecific signal in the Chd3 blot and arrow indicates the specific band. [file 13072_2022_448_MOESM3_ESM.pdf]

Figure S4

**A**

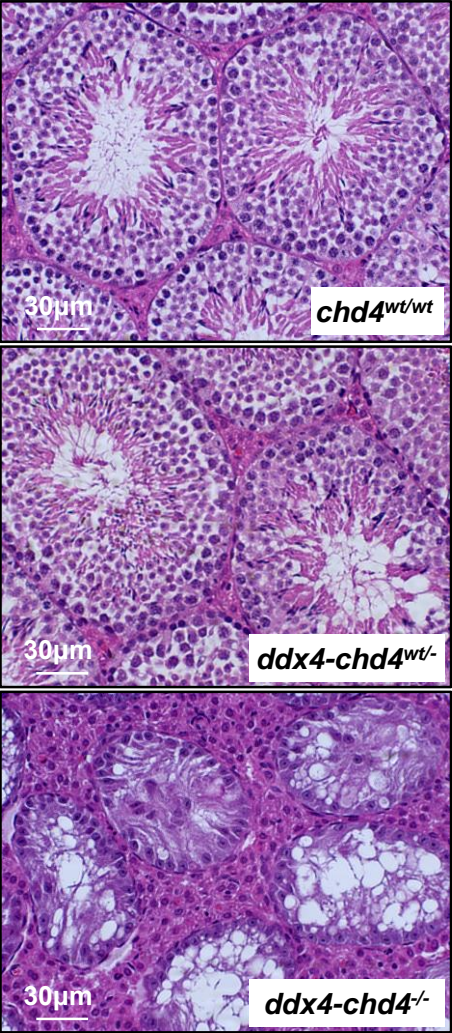

**B**

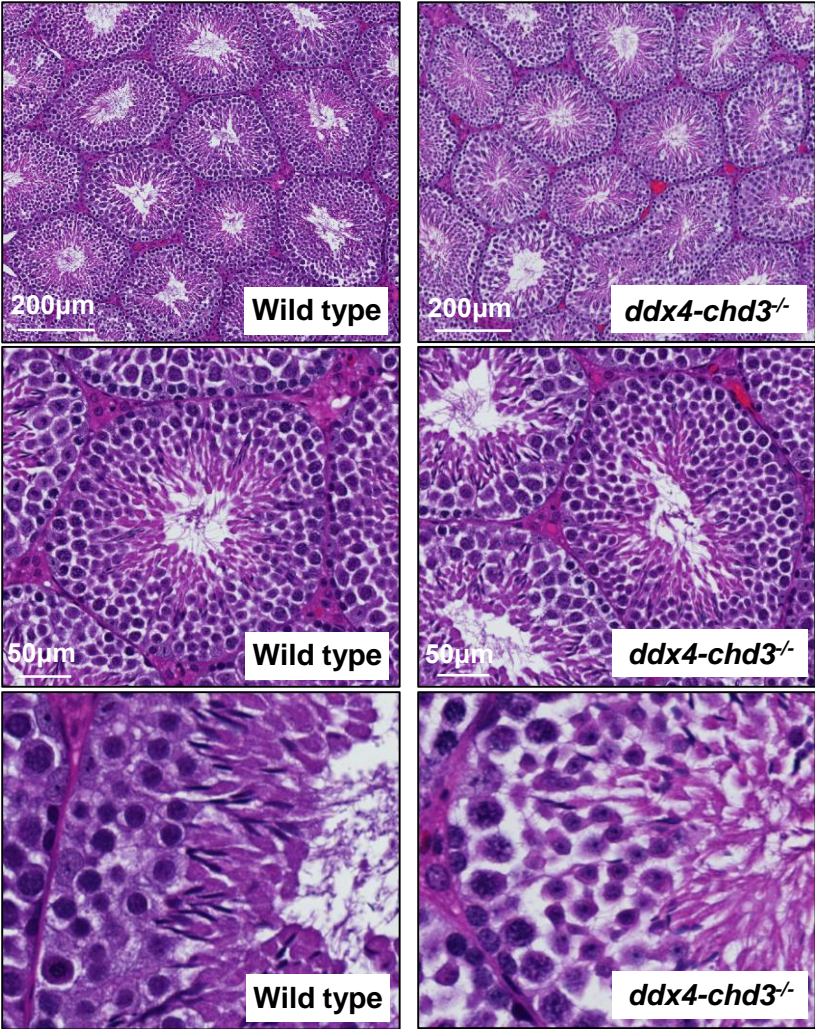

Supplement: Supplementary file 4 — Additional file 4: Figure S4. Ablation of both chd4 alleles are required for a Sertoli-only phenotype and Chd3 is dispensable for gametogenesis. A Histological sections of 2 months of old mice testis stained with H&E shows no differences between homozygous (chd4wt/wt) and the heterozygous (ddx4-chd4wt/−) mice controls, while ddx4-chd4−/− mouse results in absence of germ cells. B H&E stained histological sections of wild type and ddx4-chd3−/− testis. No differences in type or number of germ cell at any stage of development are observed between wild type and ddx4-chd3−/− knockouts. [file 13072_2022_448_MOESM4_ESM.pdf]

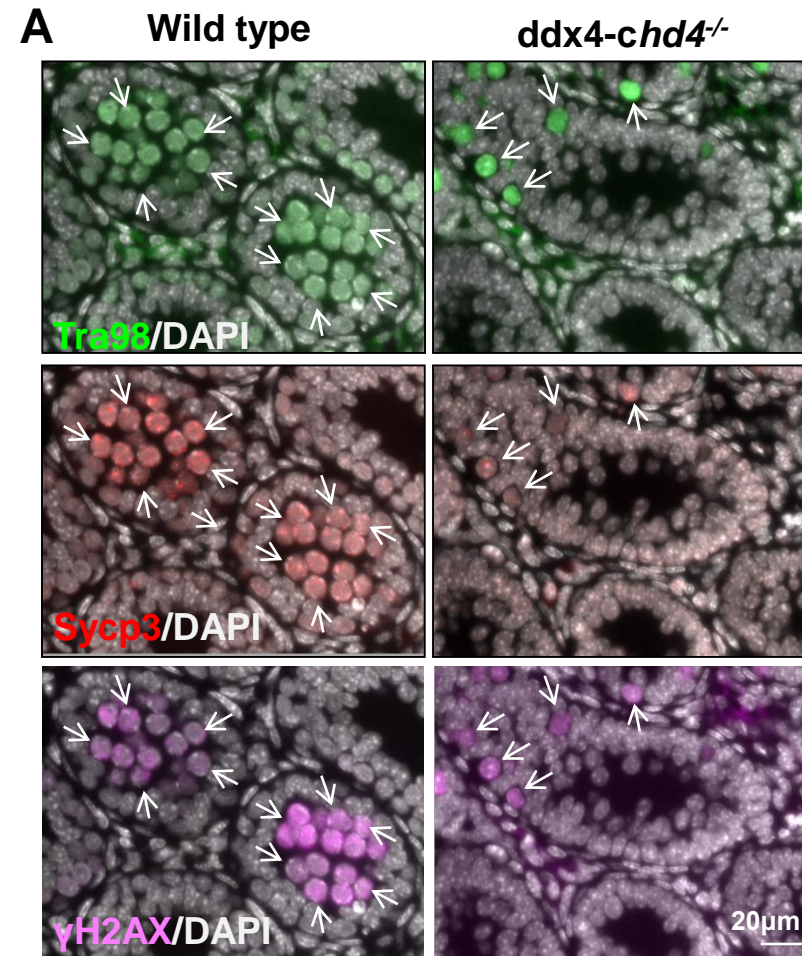

Figure S5

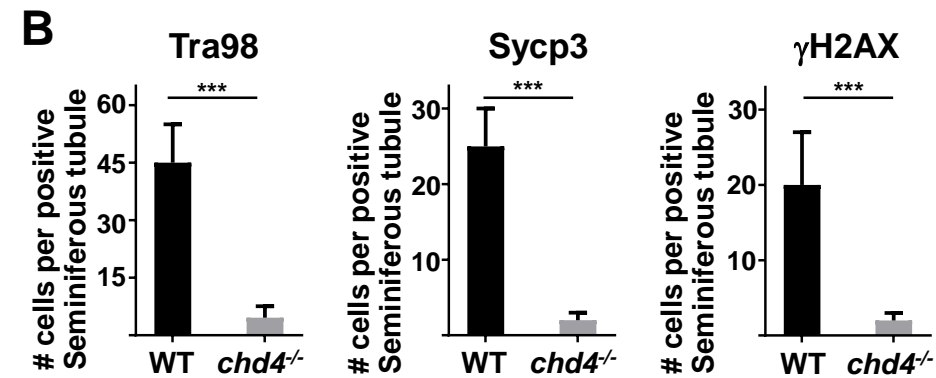

Supplement: Supplementary file 5 — Additional file 5: Figure S5. Chd4 deletion results in deficient spermatogonia cell development and near absence of spermatocytes. A Histological sections of 9 dpp wild type and ddx4-chd4−/− testis showing seminiferous tubules immunolabeled with Tra98 (a marker of germ cells) and Sycp3 and γH2AX (markers of primary spermatocytes). Arrows indicate examples of positive cells. B Quantitation of cell number per positive tubule shown in A. Tra98 positive cells in wild type (45 ± 10, n = 66) and ddx4-chd4−/− (4.6 ± 3, n = 60, P < 0.0001, Student t test) mice. Sycp3 positive cells in wild type (25 ± 5, n = 45) and Ddx4-Chd4−/− (2 ± 1, n = 45, P < 0.0001, Student t test) mice. γH2AX positive cells in wild type (20 ± 7, n = 38) and ddx4-chd4−/− (2 ± 1, n = 38, P < 0.0001, Student t test) mice. [file 13072_2022_448_MOESM5_ESM.pdf]

Figure S6

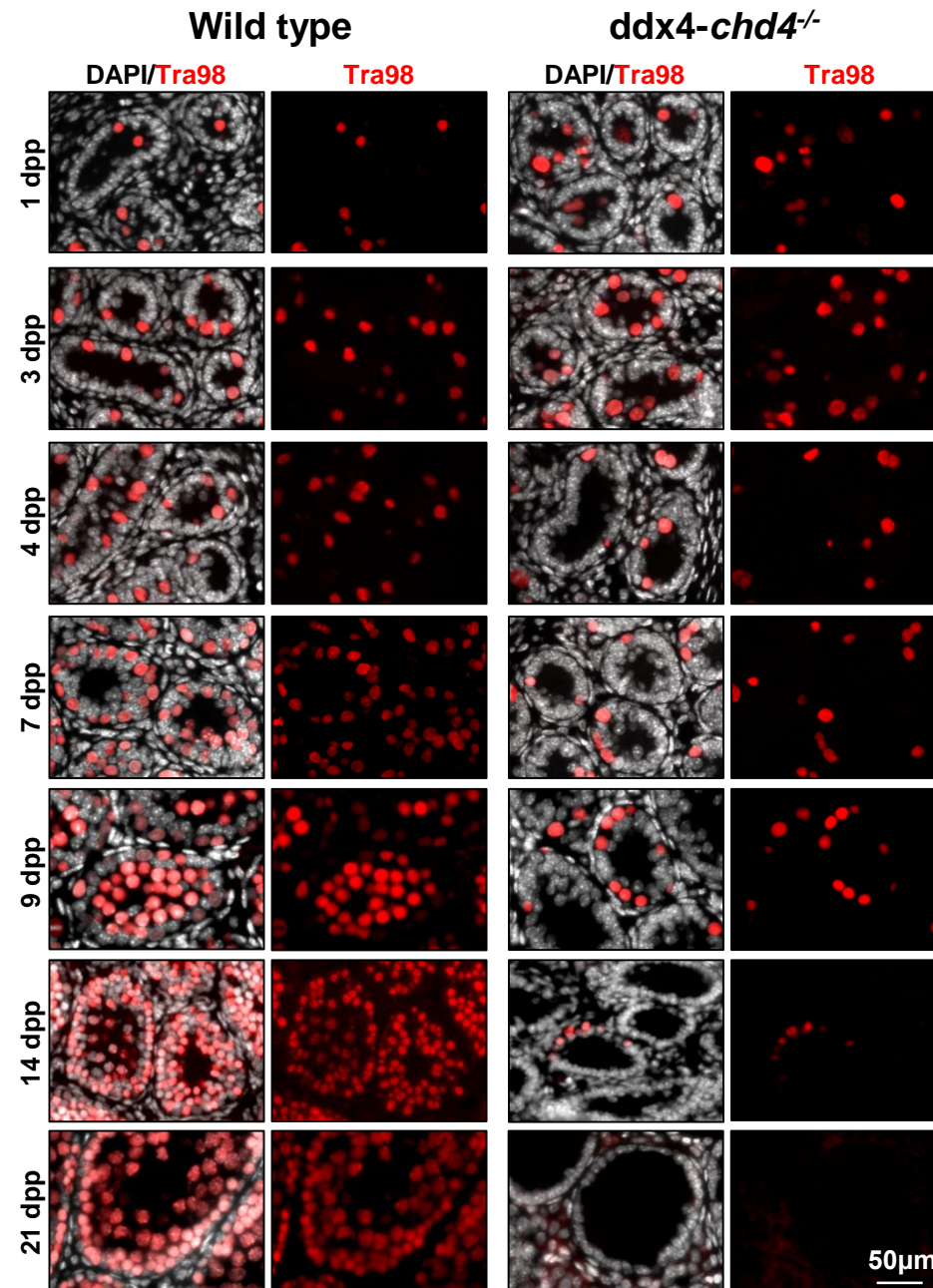

Supplement: Supplementary file 6 — Additional file 6: Figure S6. Deficient spermatogonia cell survival and differentiation in chd4−/− mice. Histological sections of wild type and ddx4-chd4−/− testis cords from 1, 3, 4, 7, 9, 14, and 21 dpp mice stained with Tra98 antibodies. See quantification in Fig. 4C. [file 13072_2022_448_MOESM6_ESM.pdf]

**A**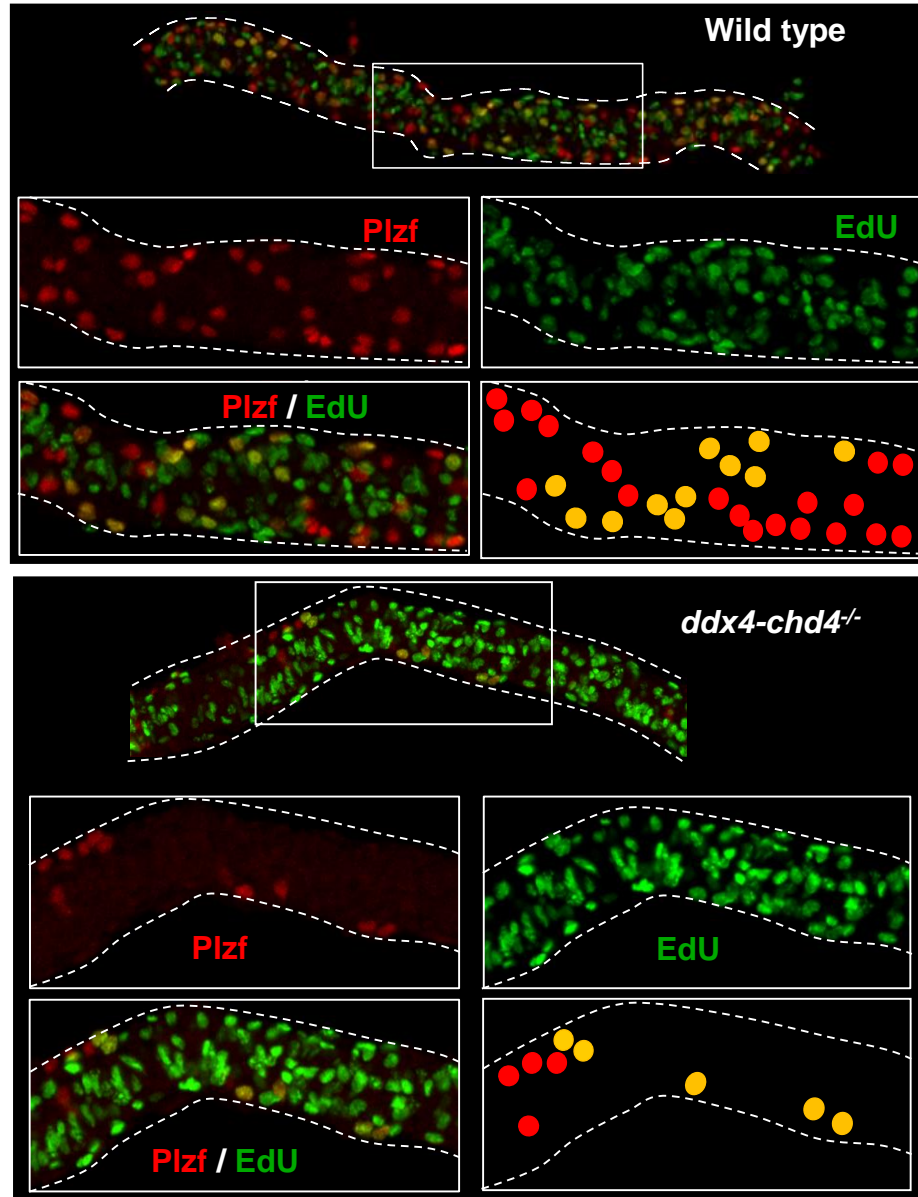

Figure S7

**B**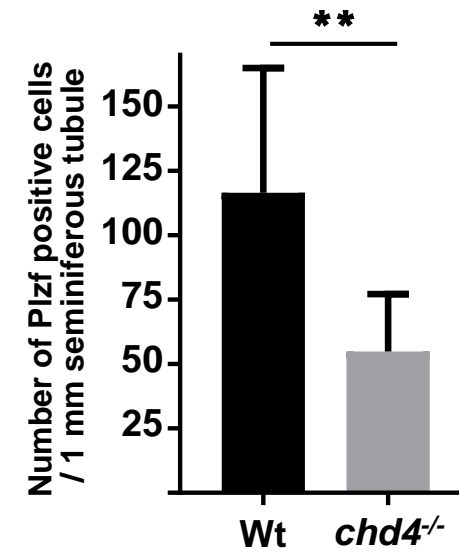**C**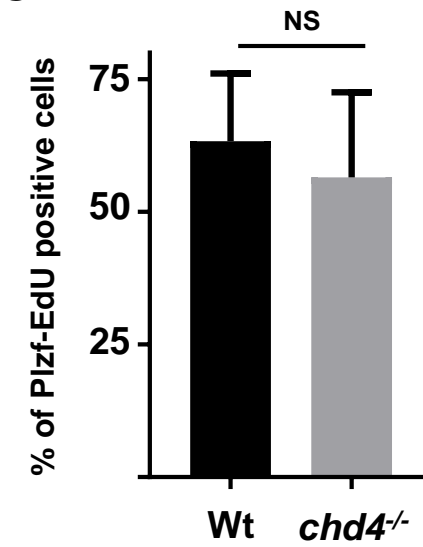

Supplement: Supplementary file 7 — Additional file 7: Figure S7. Deficient spermatogonia cell survival in chd4−/− knockout mice. A Immunostaining of whole mount seminiferous tubules reveals loss of spermatogonia (Plzf) in 4 dpp ddx4-chd4−/− mice. EdU was used to mark proliferating cells. B Quantitation of number of Plzf positive cells per mm of seminiferous tubules [wild type (116 ± 48) and ddx4-chd4−/− (54 ± 22), P < 0.0011, student t test]. Number of cells corrected by total length of seminiferous tubule analyzed in wild type versus chd4−/− mutants. A total of 7.36 mm (833 cells, wild type) and 10.15 mm (478 cells, ddx4-chd4−/−) seminiferous tubule length were counted using four different mice. C Percentage of proliferative spermatogonia cells (Plzf+/EdU+) in wild type and ddx4-chd4−/− (wild type (63% ± 13%) and ddx4-chd4−/− (56% ± 16), P < 0.2816, Student t test). [file 13072_2022_448_MOESM7_ESM.pdf]
