# Supplementary material for: Mouse Chd4-NURD is required for neonatal spermatogonia survival and normal gonad development
Source: Epigenetics Chromatin. 2022 May 14;15:16. doi: 10.1186/s13072-022-00448-5 (PMC9107693; doi:10.1186/s13072-022-00448-5)
Supplement: Supplementary file 11 — Additional file 11: Table S4. List of antibodies used in this work. [file 13072_2022_448_MOESM11_ESM.docx]

**Table S4**

**Antibodies table**

| Antibody | Source | Western blot dilution | Immuno-labeling dilution |
| --- | --- | --- | --- |
| Sox9 | EMD Millipore, AB5535 | 1:3000 | 1:500 |
| Stra8 | AbCam, ab49602 | 1:1500 | 1:500 |
| Tra98 (Gcna1) | AbCam, ab82527 |  | 1:200 |
| Plzf | Santa Cruz, sc-28319 | 1:50 | 1:50 |
| Sycp3 | AbCam, ab97672 | 1:1000 |  |
| γH2AX | Millipore, 05-636 |  | 1:1000 |
| Chd4 mAb | AbCam, ab70469 | 1:1000 | 1:300 |
| Chd4 pAb | Active Motif, 39289 | 1:1500 | 1:500 |
| Hdac2A | AbCam, ab12169 | 1:1000 |  |
| Chd3 | Bethyl, A301-220A | 1:750 |  |
| Mta1 | Bethyl, A300-280A-T | 1:1000 |  |
| Rbbp4 | Bethyl, A301-206A-T | 1:1000 |  |
| Rbbp7 | Bethyl, A300-958A-T | 1:1000 |  |
| Mbd2 | Bethyl, A301-633A-T | 1:1000 |  |
| Lamin B | AbCam, 16048 | 1:2500 |  |
| α-tubulin | Proteintech, 66031-1-Ig | 1:5000 |  |
| Dmrt1 | Santa Cruz, sc-377167 (A-9) |  | 1:100 |
| Ddx4 | AbCam, 13840 |  | 1:400 |
